# Virulence Associated Morphology of Different Strains of *Melissococcus plutonius*, a Brood Pathogen of Honey Bees

**DOI:** 10.1111/1758-2229.70369

**Published:** 2026-06-03

**Authors:** Oleg Lewkowski, Gerd Hause, Silvio Erler

**Affiliations:** ^1^ Animal Ecology, Institute of Biology Martin‐Luther‐University Halle‐Wittenberg Halle (Saale) Germany; ^2^ Institute for Bee Protection, Julius Kühn Institute (JKI) – Federal Research Centre for Cultivated Plants Braunschweig Germany; ^3^ Electron Microscopy Lab, Biocenter Martin‐Luther‐University Halle‐Wittenberg Halle (Saale) Germany; ^4^ Zoological Institute, Technische Universität Braunschweig Braunschweig Germany

**Keywords:** *Apis mellifera*, brood disease, cell morphology, cell separation, European foulbrood, virulence

## Abstract

The causative agent of European foulbrood, *Melissococcus plutonius* , kills young larvae of honey bees. Although, several clonal complexes have been studied, the specific role of bacterial phenotype regarding bacterial morphology and other physiological parameters are less well understood. Insights from prominent bacterial pathogens (e.g., *Enterococcus faecalis* ) have revealed that features like chain formation and separation play a critical role in the infection process and the course of bacterial diseases. Here, we explored the diversity in cell morphology and growth behaviour of three *M. plutonius* strains employing electron microscopy of larvae faeces and cultured bacteria, light microscopy on cultured bacteria and expression analysis of glycan‐binding protein candidate genes. We found cell morphological differences of strains from the digestive tract of infected larvae and potentially associated expressional differences of several candidate genes. A growth assay in culture medium resulted in distinct chain length profiles of tested strains. An experimental transfer of supernatant from the highly virulent (49.3) to a less virulent strain (119) consistently led to cell separation suggesting a regulatory compound released from the highly virulent strain. Our results illustrate the mechanism of cell separation and the formation of individual cocci and short chains, potentially shedding light on the underlying mechanisms.

## Graphical abstract

This study explores the diversity in cell morphology and growth behaviours of three *Melissococcus plutonius* strains, the causative agent of European foulbrood, employing electron microscopy and expression analysis of glycan‐binding protein candidate genes. Our results illustrate the mechanism of cell separation and the formation of individual cocci and short chains, potentially shedding light on the underlying mechanisms behind these observed morphological shifts.

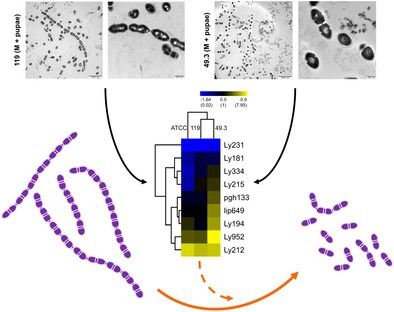

## 1 Introduction

European foulbrood (EFB) is a major bacterial brood disease of the Western honey bee *Apis mellifera* and other species of honey bees. Virulent strains of the causative agent *Melissococcus plutonius* effectively infect young larvae and may kill them in a course of days (Forsgren 2010). A variety of strains and clonal complexes have been identified employing conventional microbiological and biochemical tools, and recently expanded by molecular methods (Djukic et al. 2018). However, the role of phenotype in strain diversity and its consequences for bacterial morphology and physiology as well as virulence have been neglected. Recently, it was shown that *M. plutonius* genetic diversity is also reflected in virulence (Grossar et al. 2020; Lewkowski and Erler 2019; Pérez‐Ordóñez et al. 2021). Besides the growth rate differences, variability in colony morphology was noted. *M. plutonius* , a member of the family of Enterococcaceae, is a Gram‐positive, non‐motile bacterium, characterized by a family‐typical morphology which grows in dyads (diplococci) and chains of variable length (Alippi 1991; Bailey 1957; White 1920). Although generally observed, its growth characteristics have never been linked to the virulence of the different strains. A closely related species, *Enterococcus faecalis* , shows similar growth patterns in macroscopic colony and microscopic cell morphology and sometimes even gets confused with *M. plutonius* (Alippi 1995; Kanbar et al. 2005). Over the last decade, there were efforts to clarify the adaptive value and mechanism of chain growth in several bacterial taxa, including Enterococcaceae. An important insight was that morphology of cell formations (units) substantially affects virulence of pathogenic *E. faecalis* strains (Gilmore et al. 2014). Salamaga et al. (2017), for example, demonstrated that chain formation and separation are important factors in the pathogenesis of virulent *E. faecalis* strains. Besides *E. faecalis* there are other examples like *Streptococcus pneumoniae* or pathogenic *Escherichia coli* , where chain formation plays an important role in infection. These findings point to a conserved mechanism and a general significance of growth variation in bacterial virulence (Rodriguez et al. 2012). In previous experiments, we observed that in liquid culture *M. plutonius* grew in longer chains (Table S1) while bacteria isolated from infected larvae were found mostly in very short chains and to a larger proportion in dyads (Figures S1 and S2). These observations led us hypothesize that this specific growth behaviour may be related to bacterial virulence.

To get an insight into this system and its mechanisms, we used different microscopic, microbiological and molecular techniques to investigate bacterial cell morphological diversity and its growth behaviour, which presumably has consequences for virulence and infectivity of different strains of *M. plutonius* (Grossar et al. 2020; Lewkowski and Erler 2019). In addition, we included assays to test for expressional activity of candidate genes that may regulate cell separation and the formation of cocci.

## 2 Experimental Procedures

### 2.1 Bacteria Cultivation

We selected three *Melissococcus plutonius* strains to represent the spectrum of chain length and virulence status for our study. These strains were previously described and utilized in our earlier work as well as in studies conducted by others (Grossar et al. 2020; Lewkowski and Erler 2019). *M. plutonius* strains 49.3 (ST3, CC3, Graubünden, Switzerland), a short‐chained and highly virulent variant, and 119 (ST 20, CC13, Bern, Switzerland), a long‐chained and low virulent variant, were originally isolated from EFB outbreaks in Switzerland (Djukic et al. 2018; Grossar et al. 2020). The type strain ATCC 35311 (ST1, CC13) originated from an isolate from the United Kingdom (Bailey and Collins 1982) is low virulent to avirulent, variable in morphology and was selected to illustrate a case of a rather non‐typical variant without ecological pressure. All strains were cultivated in liquid medium consisting of 2 g/L sucrose, 10 g/L glucose, 13.5 g/L KH_2_PO_4_/K_2_HPO_4_ (1:1), 1 g/L L‐cysteine hydrochloride, 10 g/L homogenized drone pupae (white eye) and 5 g/L yeast extract for pupae medium (MCP), or no homogenized pupae and 10 g/L yeast extract for standard basal medium (MC), adjusted to pH 6.6 and incubated at 35°C with 10% CO_2_. Cultures were grown for 3 days until saturation unless stated otherwise. Cultivation success and bacteria species were confirmed according to Erler et al. (2014) and Budge et al. (2025).

### 2.2 Acquisition and Processing of Images for Basic Bacteria Chain Length Determination

For each strain and medium, we inoculated three replicates of 50 mL tubes with 10 μL of stock culture. The tubes were incubated for 3 days until the culture reached saturation, as described previously. Three samples of 2 μL per replicate were examined on a microscopic slide and two images per sample were taken on an OLYMPUS CX41 microscope mounted with an OLYMPUS Camera SC30 with 200× magnification (119, ATCC 35311) or 400× (49.3). For every single image, 25 bacterial units were measured using a grid with a square size side length of 97 μm. Images were processed with a Macro in Fiji (ImageJ version 2.0.0‐rc‐61/1.51n) using BioVoxxel tools. Subsequently, the chain perimeter from selected samples was automatically determined with binary images generated with the automatic thresholding function. Ultimately, we used the perimeter data to calculate the chain length (see details in Table S2). Preceding the final length calculations, we obtained conversion factors from acquired perimeter values to account for growth condition and strain‐related cell diameters differences (see Supporting Information for details).

### 2.3 Statistics

Variance in bacteria chain length within and among treatment groups and controls was compared by non‐parametric Scheirer–Ray–Hare tests and post hoc Dunn's tests with Bonferroni correction for multiple pairwise comparisons using the R packages *rcompanion* (version 2.3.0) and *FSA* (version 0.8.24).

### 2.4 Transmission Electron Microscopy

To get a better understanding of *M. plutonius* morphological variability and chain formation, two types of samples were prepared: (1) Liquid bacterial cultures of the strains 49.3, 119 and ATCC 35311 cultivated (to full saturation) in MCP and MC medium. (2) Faeces samples, pooled from three defaecating larvae, infected with the different *M. plutonius* strains, one group fed with *P. alvei* and uninfected controls (Lewkowski and Erler 2019). Both samples were fixed with 3% glutaraldehyde (Sigma‐Aldrich) in 0.1 M sodium cacodylate buffer (SCP, pH 7.2) for at least 4 h at room temperature. After fixation, the cells were immobilized with 4% agar in SCP, rinsed in SCP and postfixed with 1% osmium tetroxide (Carl Roth) in SCP for 1 h at room temperature. Subsequently, the samples were rinsed with water, dehydrated in a graded ethanol series (10%, 30%, 50%, 70% ethanol containing 1% uranyl acetate (Chemapol), 70%, 90%, 2 × 100%, for 30 min each), infiltrated with epoxy resin according to Spurr (1969) and polymerized at 70°C for 24 h.

Ultrathin sections (50 nm) were made with an Ultracut R ultramicrotome (Leica). Sections were post‐stained with uranyl acetate and lead citrate in an EM‐Stain apparatus (Leica) and subsequently observed with an EM 900 transmission electron microscope (Carl Zeiss Microscopy) operating at 80 kV. Micrographs were taken with a Variospeed SSCCD camera (Tröndle).

### 2.5 Autolysis Experiment

Cell signalling, including quorum sensing, is a major feature of bacterial communities. In numerous bacterial species, for example, *E. faecalis* , chain length as well as cell separation was shown to be regulated by specific enzymes, named autolysins. Following our observations of the growth of *M. plutonius* and in regard to its close relatedness to *E. faecalis* , we assumed a similar mechanism. Such mechanisms are essential for the pathogenesis and the specific modes and timing of infection and hence may vary in strains with different virulence as described before.

Therefore, we hypothesize that one or multiple compounds released into the surrounding medium would act as a signalling agent, an active compound, or even both, and potentially promote a breakdown of chains with cell separation through initiation of an intra‐cellular or external mechanism. This hypothesis was tested using strain 49.3 (shortest chains, potentially high production of autolysins) and strain 119 (longest chains, potentially low production of autolysins) by interchanging their respective cultural overflow (supernatant), presumably containing active molecules that are involved in cell separation.

Bacterial cultures were grown in MC medium as described above. After 3 days, 1000 μL per 1.5 mL reaction tube of bacterial cultures from strain 49.3 and 119 were centrifuged at 20,817× *g* (14,000 rpm) for 10 min and the filter‐sterilized supernatant (membrane pore size: 0.22 μm, TPP Techno Plastic Products AG) was used for the experiment. Pelleted bacterial cells were washed with distilled water and centrifuged for a second time (same conditions). Water supernatant was removed and bacterial cells were dissolved in filter‐sterilized supernatant from the same culture (control 1: 49.3–49.3, 119–119) or from the other culture (treatment: 49.3–119, 119–49.3). One millilitre of the remaining material of the respective cultures was pelleted and re‐dissolved in their own supernatant as untreated controls (control 2: 49.3, 119). This procedure was repeated for three replicates each and cultures were incubated for 24 h before examining chain lengths (see previous section).

### 2.6 Expression of Potential Virulence and Chain Separation Related Genes in *M. plutonius*

Bacterial cultures were grown in MCP medium (50 mL tubes) to saturation (3 days) and 1 mL was centrifuged at 20,817× *g* for 3 min at 4°C. Supernatant was discarded and pellets were washed in 1 mL DEPC‐water. Centrifugation was repeated for 2 min and the resulting pellet was dissolved in 100 μL TE‐buffer containing 20 units lysozyme (Sigma‐Aldrich), and incubated for 10 min at 37°C. Subsequently, bacterial RNA was extracted using the NucleoSpin RNA purification kit (Macherey‐Nagel) according to the manufacturer's protocol, including an on‐column rDNAse digestion step. RNA integrity was verified on 1.6% denaturing formaldehyde agarose gels in MOPS buffer (PUFFERAN, Carl Roth). Five microlitre of sample were mixed with 5 μL RNA loading dye (2×, Fermentas) and denatured for 10 min at 70°C before loading on the gel.

cDNA synthesis was performed with 500 ng of purified RNA, random hexamer primers (Fermentas) and M‐MLV Reverse Transcriptase (Promega) according to the manufacturer's protocols. RNA and cDNA concentrations were determined on a Nanodrop 1000 (Thermo Fisher Scientific) spectrophotometer.

Real‐time qPCR primers for bacterial autolysins, cell wall lytic enzymes, peptidoglycan hydrolases and the bacterial reference gene *atlA1* (endo‐β‐*N*‐acetylglucosaminidase) were designed using Primer3 and Primer‐BLAST (NCBI) (Table S3). The reference gene had a stable Cq value among all groups of 23.74 ± 0.9 (mean ± SD) as well as in all pre‐trials (Cq SD < 0.5). For primer selection, optimization and estimating PCR efficiency, a dilution series (1:10 to 1:10,000) was run for each primer pair, and the PCR products were analysed for specificity and expected fragment sizes on a QIAxcel automatic capillary electrophoresis system (Qiagen).

qPCRs were run using the CFX Connect Real‐Time PCR Detection System (Bio‐Rad), using SensiMix SYBR No‐ROX kit (Bioline) and gene specific primers (0.3 μM each, Table S3) in triplicate 10 μL reactions (including 1 μL 1:10 diluted cDNA template), with the following protocol: initial denaturation at 95°C for 10 min, 40 cycles of 95°C for 15 s, 60°C for 20 s and 72°C for 20 s, final elongation at 72°C for 30 s. The melting curve of all amplicons was measured from 50°C to 98°C, every 5 s, with 1°C increment and checked for consistency.

## 3 Results and Discussion

### 3.1 Strain Related Chain Length Differences

**FIGURE 1:**
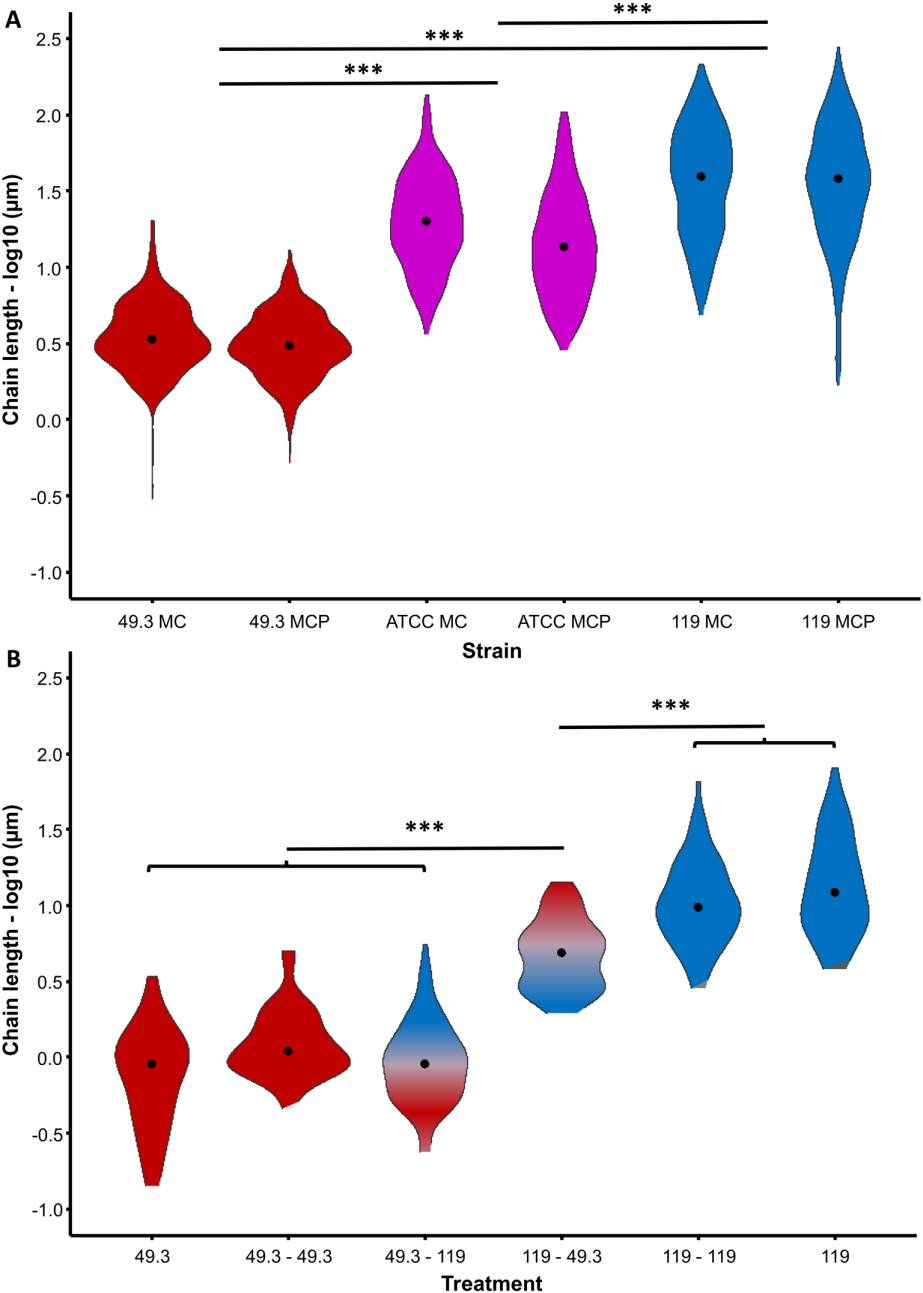
Chain lengths of several *M. plutonius* strains (ATCC 35311, 49.3 and 119) under various growth conditions. (A) Strains grown in standard control medium (MC) and in medium containing homogenized honey bee drone pupae (white eye; MCP) (Lewkowski and Erler 2019). (B) Strain 49.3 and 119 grown in control medium (49.3, 119), in medium containing the supernatant of the same strain (49.3–49.3, 119–119) or medium containing the supernatant of the opposite strain (49.3–119, 119–49.3). Y‐axes are log‐scaled. (***p < 0.001).

The chain lengths of the three different *M. plutonius* strains did not differ significantly among their replicates (SRH‐test, *H* = 3.32, df = 2, *p* = 0.19). However, we clearly found significant differences in chain length between *M. plutonius* strains (SRH‐test, *H* = 1734.41, df = 2, *p* < 0.001), with strain 119 having the longest chain length, strain ATCC 35311 with medium‐long chains, and strain 49.3 having the shortest chain length (Figures 1A, S3 and Table S4). Across all *M. plutonius* strains, bacteria chains grown in MC medium were longer than in MCP medium (SRH‐test, *H* = 22.32, df = 1, *p* < 0.001).

While previous research has highlighted the diversity in genome composition (Djukic et al. 2018) and virulence‐related host–pathogen interactions (Grossar et al. 2020; Lewkowski and Erler 2019) of *M. plutonius* and its host, *A. mellifera* , our study reveals a phenotypic variation among *M. plutonius* strains. Earlier studies presented differences in chain length and appearance in chains of pairs of cocci among diverse strains from Brazil, China, Japan and United Kingdom, however, without focusing on length variation and relevance for pathology (Allen and Ball 1993; Arai et al. 2012). Based on our observations and the experimental data presented in this work, we suggest a general relationship of phenotypic differences in chain length and virulence in *M. plutonius* . Like described previously, chain length is potentially linked to growth dynamics and functional morphology of bacteria. This is also supported by observations in other studies. Reports on several strains indicate that *M. plutonius* generally maintains low average cell counts, typically between three and five cells per chain and for instance DAT561 as highly virulent strain with the lowest number of cells per chain (Okamoto et al. 2022; Takamatsu et al. 2020). While Arai et al. (2012) observed a long‐chained strain DAT606 (Japan, CC3, ST3) that was not killing infected larvae, a highly virulent strain from Canada (BHB413, Fraser Valley, British Columbia, ST19, CC12), in contrast, was characterized by short chains of lanceolate cocci (Wood et al. 2020).

However, these morphological traits are highly plastic and depend on culture conditions. It is important to note that the longer the chain length, the fewer bacterial colonies were observed on agar plates. This finding might be relevant for phenotypic pre‐screening. A recent study confirmed that the average number of cells per chain is strongly dependent on culture conditions: agar plate versus broth, basal medium versus KSBHI and also on timing: mostly lower cell numbers per chain for longer cultivation duration (Kitamura et al. 2025). In broth cultivation, CC13 strains formed the longest chains with the highest numbers of cells per chain. CC12 strains, on the other hand, had the shortest chains, while CC3 strains were intermediate in length. This is in concordance with the observations for CC13 and CC3 strains in our own studies.

### 3.2 Chain Morphology and Microscopic Details

**FIGURE 2:**
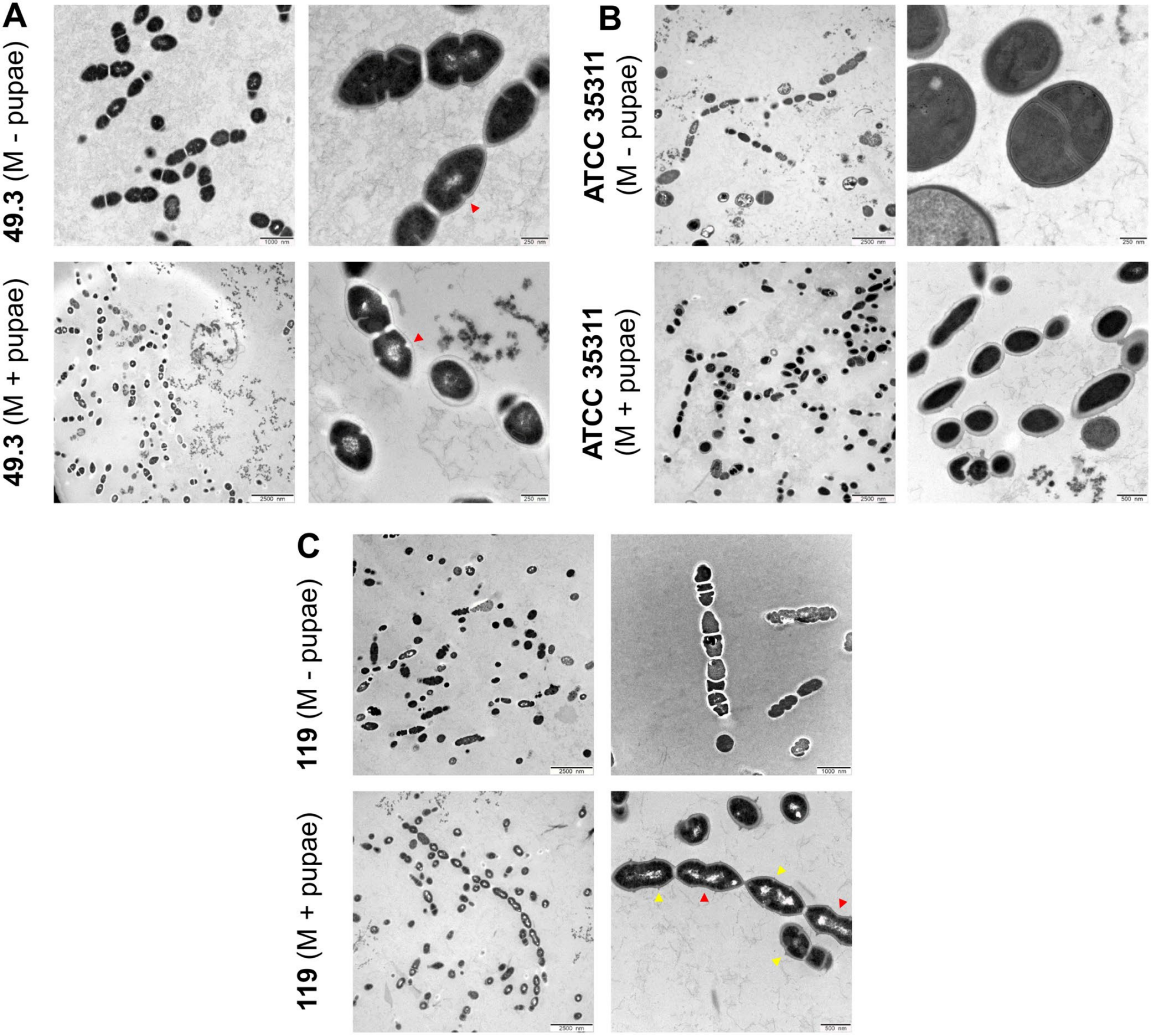
Transmission electron microscopy pictures of (A) short‐chain strain *M. plutonius* 49.3, (B) medium‐chain strain *M. plutonius* ATCC 35311 and (C) long‐chain strain *M. plutonius* 119, grown in vitro in standard medium containing homogenized honey bee drone pupae (white eye, MCP) (Lewkowski and Erler 2019) and without (M—pupae, MC). (red arrow heads point to incomplete cell division, yellow arrow heads point to spike‐like ridges).

Transmission electron micrographs clearly show the variability in cell forms and chain lengths of the three different *M. plutonius* strains (Figure 2). Some cell properties seem to be strain‐specific and most likely related to adaptations to their environment. Bacterial strains 49.3 and 119 have a family characteristic cell morphology and cell separation features, specifically the leading chain formation resulting from incomplete division also found in *E. faecalis* .

The basic unit of chain formation in strain 49.3 was a diplococci‐like form, without obvious signs of long chain formation, irrespective of the cultivation medium (Figure 2A). This is the regular form of several *M. plutonius* strains described in earlier work (Dicks and Holzapfel 2015; White 1920).

We found that single cells and medium‐long chain formations of the reference strain ATCC 35311 had very variable morphology. Cells often appear malformed and chains seemed to divide in random places, some of them very quick others very slow (Figure 2B). Further, many spherical‐shaped cells can be observed (Figure 2B, higher magnification). The varied shapes of cells may be a result of decades of laboratory cultivation and a lack of environmental selective pressure from culture media. However, comparing transmission electron micrographs of the current study with scanning electron micrographs of the reference strain grown in culture medium (Hannes Beims, person. comm., Figure S4; Alippi 1991), the medium‐long chains are the same phenotype of the strain but cell morphology differs. This might result from the different fixation methods, temporal variability in re‐cultivation frequency in the different labs, or is based on minor differences in the composition of the bacteria growth medium.

The cell walls of the very long‐chained strain 119 had spike‐like ridges on the outside of the wall (also called wall bands), which presumably originate from cell division and post‐fission movement of the outer layer during further wall growth (Figure 2C). This morphological feature was not observed for the other strains. Previous studies described such spike‐like ridges as scars, which are remnants of the outer part of the cell wall after cell division by the snapping mechanism (Blom and Heltberg 1986). Scars are indicators of the outer layer rupture points and can be seen as locations of septum formation (Krulwich and Pate 1971). For a detailed description of the cell separation model for *E. faecalis* see Higgins and Shockman (1970).

Longer cellular chains appear to generate a signal that may provide an adaptive advantage for chain separation in the host. For *E. faecalis* , for example, it was proposed that longer chains are a morph of dissemination and systemic infections (Gilmore et al. 2014). While long‐chained forms were often found to maintain a biofilm, shorter forms like single cells and diplococci are efficient in evading cellular immune responses (Salamaga et al. 2017). Previously, we found that honey bee larvae express specific immune and metabolic responses to infections with *M. plutonius* (Lewkowski et al. 2022). Using larvae infection assays, we could show that medium‐ and long‐chained strains might be eliminated more effectively by the host's defence system compared to the short‐chained strain 49.3, as indicated by a much higher number of degenerated cells and empty cell envelopes in faecal samples (Figures S1 and S2). The observed host defence (by cell killing) was pathogen‐specific, as controls infested with *Paenibacillus alvei* resulted in growing bacteria (Figure S1). In conclusion, while no specific advantages for different morphotypes in *M. plutonius* are known until now, our observations suggest a functional significance of chained and single‐celled (and diplococci) forms.

### 3.3 Autolysis Experiment

Statistical analysis revealed an overall effect of treatment (SRH‐test, *H* = 307.79, df = 5, *p* < 0.001) but not replicates within treatment groups (SRH‐test, *H* = 0.74, df = 3, *p* = 0.69) or any replicate × treatment interaction (SRH‐test, *H* = 1.437, df = 8, *p* = 0.99). Treatment of long‐chained strain 119 (119–49.3) resulted in significantly reduced chain lengths after 24 h of incubation, compared to the controls (119–119, 119) (Dunn tests, Table S5), and therefore points to a cell separation inducing agent in the supernatant of the short‐chained strain 49.3 (Figures 1B and S3, Table S6). Vice versa, treating strain 49.3 with supernatant of strain 119 did not result in any significant chain length change compared to the controls (49.3–49.3, 49.3) (Dunn tests, Table S5).

So far, a specific agent responsible for inducing chain separation, that is released into the medium, has yet to be identified. In *E. faecalis*, two proteins (AtlA, GelE) were described to be involved in the mechanism controlling chain length. The AtlA protein digests the septum and is required for cell separation. Its depletion or lack of processing results in the formation of long chains (Mesnage et al. 2008). AtlA function is regulated via proteolytic activation by the gelatinase GelE (M4 family metallopeptidase coccolysin). For efficient AtlA‐mediated cell division, *N*‐terminal cleavage is required (Stinemetz et al. 2017). GelE secretion into the supernatant has been shown to impact the degradation of misfolded surface proteins, pheromone levels and enhances autolysis, whereas its disruption led to increased cell chain length of the diplococcal state of *E. faecalis* (Waters et al. 2003). This shows that post‐translation modification is mandatory for septum localization and subsequent cell separation into smaller units. However, screening *M. plutonius* genomes showed that *autolysin* ( *E. faecalis* , loci: WMS_RS07300, WMS_RS08220, WMS_RS12985), *gelE* ( *E. faecalis* , WMS_RS12215), a DUF5776 domain‐containing protein ( *E. faecalis* , WMS_RS13420) and *E. faecalis* *lysozyme* (WMS_RS15690) had no homologous genes or proteins in the genomes (using NCBI BLASTN and Quick BLASTP).

### 3.4 Expression of Potential Virulence and Chain Separation Related Genes in *M. plutonius*

**FIGURE 3:**
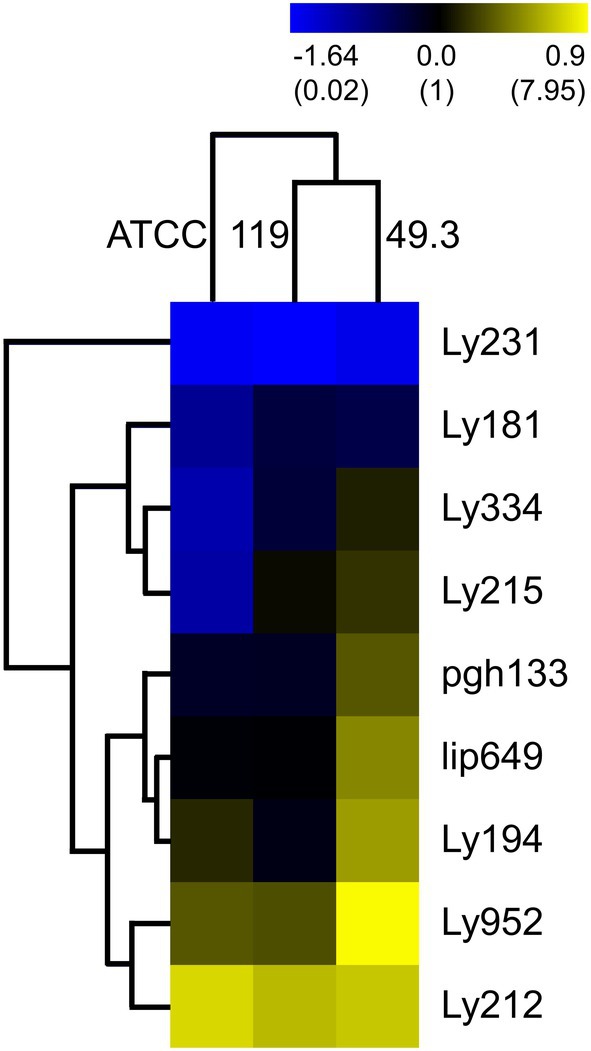
Heatmap showing gene expression of potential virulence and chain separation related genes in several *M. plutonius* strains (ATCC 35311, 49.3 and 119). Log transformed expression values were visualized using hierarchical clustering analysis with optimized gene and sample order, Euclidean distance, and average linkage clustering by the software MultiExperiment Viewer (MeV) version 4.9. Normalised gene expression values are shown in brackets.

Comparing relative gene expression among the three different strains on the third day of growth, strain 49.3 exhibited the strongest expression of the majority of the candidate genes and coincided with the onset of a notable cell separation in this strain. The other two strains (119 and ATCC 35311) differed partially from each other. The medium‐long chained strain ATCC 35311 had higher relative gene expression values for a subset of genes than 119 (Figure 3, Table S7). Among the nine tested candidate genes, we identified two major clusters. The first cluster is marked by the down‐regulation of three genes (Ly181, Ly334 and Ly215) in the ATCC strain when compared to the other strains in our analysis. This suggests these genes may be less active in the ATCC strain. The second cluster, which includes the genes Ly194, Ly952, lip649 and pgh133, shows a pattern of up‐regulation in the 49.3 strain relative to the others. A particularly notable finding within this group is the highest expression of the candidate gene Ly952 (LysM domain protein) in the 49.3 strain. The distinct and correlated expression patterns observed in both clusters suggest that transcriptional timing is most likely linked to the developmental stages of the bacteria and their characteristic chain lengths. Two other genes, Ly231 and Ly212, showed no strong differences in expression across any of the strains (Figure 3).

Numerous genes might be involved in chain separation or the formation of diplococci, whereas for most of our target genes there are currently unknown or uncertain functions. Several target gene products contain a LysM motif, which is involved in *N*‐acetylglucosamine recognition and binding (Mesnage et al. 2014), or belong to the *N*‐acetylmuramoyl‐L‐alanine amidase family. *N*‐Acetylmuramoyl‐L‐alanine amidases are mostly involved in bacterial cell development, where they play an important role in peptidoglycan biosynthesis and autolysis. These functions are controlling the speed of bacterial multiplication and hence the invasion of a host. LysM motif containing proteins can have a plethora of different functions and are found across different kingdoms (Buist et al. 2008). Binding of peptidoglycans can, for instance, involve defensive or autolytic cell separation in bacteria, defences against fungi, or recognition, binding and lysis of insect chitin.

Several genome and infection studies consistently demonstrate that the virulence mechanism in *M. plutonius* is complex. Virulence was correlated with growth dynamics and presence of the pMP19 plasmid carrying *melissotoxin A* (Djukic et al. 2018; Grossar et al. 2020). However, in another study, *melissotoxin A* had a variable impact on virulence among strains (Nakamura et al. 2020). A population‐wide screening revealed a temporal increase in the occurrence of *melissotoxin A*, suggesting that this gene promotes the dispersal of *M. plutonius* strains carrying this plasmid (Grossar et al. 2023). On the other hand, many putative virulence factors (e.g., endo‐alpha‐*N*‐acetylgalactosaminidase, enhancin and epsilon toxin; Djukic et al. 2018) were identified from genomic studies but need to be confirmed in infection studies using genetically modified strains. Notably, proteins involved in peritrophic membrane degradation like Enhancin, Chitin‐binding domain‐containing protein, and Endo‐α‐*N*‐acetylgalactosaminidase are not indispensable virulence factors for the highly virulent strain DAT 561 (Nakamura et al. 2021).

In general, bacteria can often alter their shape in response to environmental cues. This plasticity allows them to adapt to different conditions within the host and optimize their interactions with host cells and surfaces (Young 2006). Enzymes which regulate the cell morphology and chain length as well as the developmental timing may have a crucial role in the pathogenic cycle of *M. plutonius* . Further, morphology may also contribute to survival in inactive states inside or outside the host and hence facilitate the spread of the pathogen.

## 4 Conclusions

The present study explored the correlation of phenotypic diversity and variation in gene expression of cell separation‐associated proteins with known differences in virulence among strains of a pathogenic bacterium infecting honey bee larvae. By integrating light and electron microscopy and gene expression profiling, our study links both timing and extent of bacterial chain separation to strain‐specific pathogenicity. In in vitro growing experiments, we associate more rapid or extensive chain separation with increased virulence. These findings provide new insights into the biology of *M. plutonius* and the interaction with its host, as they suggest that processes of bacterial chain separation potentially represent a functionally important mechanism in pathogenesis. Separate clusters of genes showed a differential and strain‐specific expression, which might be indicative of shifts in pathogen development and host colonization. Our results also indicate that control of bacterial cell separation and its timing may offer promising avenues for mitigating the severity of EFB disease in honey bee colonies.

Our results emphasize the role of morphological features like chain length and its regulatory mechanisms in the pathogenicity of *M. plutonius* strains. The approach adopted here lays the groundwork for future investigations into cell differentiation and virulence regulation in *M. plutonius* infections and its significance in host–pathogen interactions. Future research should aim to extend these observations to in situ experiments with targeted morphological and genetic manipulations of different pathogen strains to further clarify the underlying mechanisms and pathways and their significance in the course of the disease.

## Author Contributions

**Oleg Lewkowski:** conceptualization, methodology, software, data curation, investigation, validation, formal analysis, visualization, writing – original draft, writing – review and editing. **Gerd Hause:** methodology, validation, visualization, writing – review and editing. **Silvio Erler:** conceptualization, visualization, funding acquisition, writing – review and editing, resources, project administration, supervision.

## Funding

This work was supported by Deutsche Forschungsgemeinschaft (ER 786/1‐1).

## Conflicts of Interest

The authors declare no conflicts of interest.

## Supporting information


**Table S1:** Overview on growth diversity and cell morphology of different *M. plutonius* strains.
**Table S2:** Bacteria chain width from perimeter of different bacterial strains (MC: standard control medium, MCP: medium containing homogenized honey bee drone pupae [white eye]).
**Table S3:** Bacteria target loci, primer sequences, melting temperatures (T_m_), PCR efficiencies, product size (bp) and target locus in the genome of *Melissococcus plutonius* ATCC 35311.
**Table S5:** Results of pairwise comparison Dunn's tests with Bonferroni corrected *p* values for the autolysis experiment (significant differences are highlighted in italic *p* values).
**Figure S1:** Transmission electron microscopy pictures of larvae faeces samples, larvae infected with *M. plutonius* (strain 49.3, 119, ATCC 35311) or feed with *P. alvei* only, and respective uninfected controls (free of bacteria). Bacteria are surrounded by food remains (mainly royal jelly) and larval gut lining and tissue.
**Figure S2:** Transmission electron microscopy pictures of larvae faeces samples, larvae infected with *M. plutonius* (strain 49.3) only, at four different magnifications.
**Figure S3:** Light microscopy of *M. plutonius* growth assays in medium with or without homogenized honey bee drone pupae (white eye) (Lewkowski and Erler 2019), and strains 49.3 and 119 grown in medium containing the supernatant of the same strain (+49.3/+119) or medium containing the supernatant of the opposite strain (+119/+49.3).
**Figure S4:** Scanning electron microscopy pictures of *Melissococcus plutonius* strain LMG20360 (=DSM 29964, ATCC 35311), at four different magnifications (A–D) (copy right by Hannes Beims and Manfred Rohde).

**Table S4:** Complete list of all basic perimeter measurements of individual bacterial units and derived calculated chain length of the different strains used in this study.

**Table S6:** Complete list of all measurements of individual bacterial units and derived calculated chain length of different treatments from the autolysis experiments in basic MC medium.

**Table S7:** qPCR data of potential virulence and chain separation related genes in *M. plutonius* .

## Data Availability

The data that support the findings of this study are available in the Supporting Information of this article.
