# Supplementary material for: Virulence Associated Morphology of Different Strains of Melissococcus plutonius, a Brood Pathogen of Honey Bees
Source: Environ Microbiol Rep. 2026 Jun 3;18(3):e70369. doi: 10.1111/1758-2229.70369 (PMC13239739; doi:10.1111/1758-2229.70369)
Supplement: Supplementary file 1 — Table S1: Overview on growth diversity and cell morphology of different M. plutonius strains. Table S2: Bacteria chain width from perimeter of different bacterial strains (MC: standard control medium, MCP: medium containing homogenized honey bee drone pupae [white eye]). Table S3: Bacteria target loci, primer sequences, melting temperatures (Tm), PCR efficiencies, product size (bp) and target locus in the genome of Melissococcus plutonius ATCC 35311. Table S5: Results of pairwise comparison Dunn's tests with Bonferroni corrected p values for the autolysis experiment (significant differences are highlighted in italic p values). Figure S1: Transmission electron microscopy pictures of larvae faeces samples, larvae infected with M. plutonius (strain 49.3, 119, ATCC 35311) or feed with P. alvei only, and respective uninfected controls (free of bacteria). Bacteria are surrounded by food remains (mainly royal jelly) and larval gut lining and tissue. Figure S2: Transmission electron microscopy pictures of larvae faeces samples, larvae infected with M. plutonius (strain 49.3) only, at four different magnifications. Figure S3: Light microscopy of M. plutonius growth assays in medium with or without homogenized honey bee drone pupae (white eye) (Lewkowski and Erler 2019), and strains 49.3 and 119 grown in medium containing the supernatant of the same strain (+49.3/+119) or medium containing the supernatant of the opposite strain (+119/+49.3). Figure S4: Scanning electron microscopy pictures of Melissococcus plutonius strain LMG20360 (=DSM 29964, ATCC 35311), at four different magnifications (A–D) (copy right by Hannes Beims and Manfred Rohde). [file EMI4-18-e70369-s004.pdf]

Supplementary material

Virulence associated morphology of different strains of *Melissococcus plutonius*, a brood pathogen of honey bees

Oleg Lewkowski[1,2], Gerd Hause[3], Silvio Erler[1,2,4]

[1]Animal Ecology, Institute of Biology, Martin-Luther-University Halle-Wittenberg, 06099 Halle (Saale), Germany

[2]Institute for Bee Protection, Julius Kühn Institute (JKI) – Federal Research Centre for Cultivated Plants, 38104 Braunschweig, Germany

[3]Electron Microscopy Lab, Biocenter, Martin-Luther-University Halle-Wittenberg, 06099 Halle (Saale), Germany

[4]Zoological Institute, Technische Universität Braunschweig, 38106 Braunschweig, Germany

Acquisition of images and bacteria chain length determination

1. Sample preparation and microscopy:

- 3 replicates (tubes) were used for data acquisition
- 3 samples of 2 µl were taken per tube and placed on a microscopic slide
- At least 2 images (size: 2048 × 1532 pixels, resolution: 200 dpi, colour depth: 24 Bit, no compression) were taken per sample on an OLYMPUS CX41 with an OLYMPUS Camera SC30 with 200× magnification (119, ATCC) or 400× (49.3)
- For every single picture 25 bacterial units were measured within a grid on a diagonal with a square size of 97 µm side length

2. Digital image processing for bacteria chain length determination:

- Start **ImageJ**
- Go to *Process/ Batch/* **Macro…** (or *Plugins/ Macros/* **Run…** for single images)

Macro workflow:

[

↘ Image to 8-Bit grey scale

↘ Pseudo flat-field correction – Blurring radius 5 Pixels

↘ Subtract Background Rolling Ball – Radius 10 Pixels + sliding paraboloid

↘ Enhance contrast → Saturated Pixels = 0.2% + Normalize

↘ Auto Threshold (Methode= Default) → **Binary Image**

]

- Go to *Analyze/ Tools/* **Grid…** (97 µm square side length)
- Go to *Analyze/ Tools/* **Synchronize Windows…**
- Select 25 cells on the diagonal of the grid (top left to bottom right) for measurements with Wand tool (already selected) and press [t] to save ROI in ROI Manager

Example of the application of chain length determination in a detail of a processed binary image of the strain 49.3 and two selected units (red, left) and a corresponding original microscopic image with the same units (yellow outline, right):

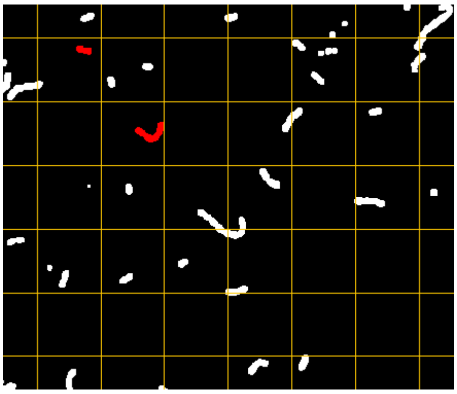

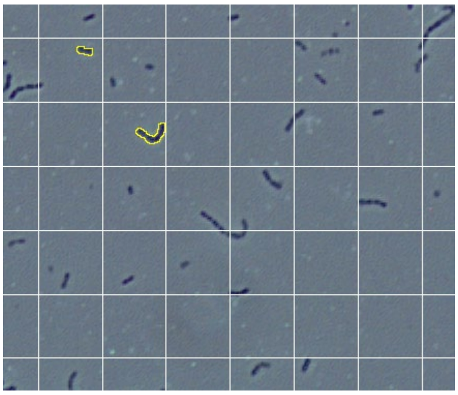

The perimeter was transformed from measured pixel values with a factor of 0.015625 (200×) and 0.03125 (400×) accounting for pixels per micrometer and magnification. Finally, bacteria unit length was calculated with the mean values of the obtained cell width (see Tab. S2):

Unit length = 0.5 × (perimeter – π × cell width) + cell width

Table S1: Overview on growth diversity and cell morphology of different *M. plutonius* strains.

Strain	Growth	Chain length
ATCC 353111	slow	long
119	medium	very long
49.3	fast	short

Table S2: Bacteria chain width from perimeter of different bacterial strains. (MC: standard control medium, MCP: medium containing homogenized honey bee drone pupae (white eye))

Strain	Medium	N	Cell width (µm)	SD (µm)
119	MC	30	1.124	0.115
119	MCP	30	1.174	0.082
49.3	MC	30	1.170	0.112
49.3	MCP	30	1.139	0.090
ATCC	MC	30	1.296	0.127
ATCC	MCP	30	1.250	0.112

Table S3: Bacteria target loci, primer sequences, melting temperatures (T_m), PCR efficiencies, product size (bp) and target locus in the genome of *Melissococcus plutonius* ATCC 35311.

Name	Sequence (5'-3')	T_m	PCR_{eff}	Size (bp)	Target	Locus
**lip649 R**	TGGAACCAACCGGGAGAATG	60	2.00	113	secreted antigen GbpB/SagA/PcsB, putative peptidoglycan hydrolase	BAK20808
**lip649 R**	TCTGTAGCTCCTGAGCCTGT	60				
**Ly181 F**	AACGGTGTCTGGCAAGTACG	60	1.99	116	phage lysin, N-acetylmuramoyl-L-alanine amidase	BAK21897
**Ly181 R**	TGCATCGAACCATCTACTGGA	59				
**Ly194 F**	GGTTTTCCGGCTTTTTCCGT	58	2.02	117	N-acetylmuramoyl-L-alanine amidase family 4, needed for cell separation and autolysis	BAK22090
**Ly194 R**	AACGTCAAAAGCACCTACGC	58				
**Ly212 F**	GTGCTGCTGCGTCATTTTCT	58	1.99	114	N-acetylmuramoyl-L-alanine amidase family 4, needed for cell separation and autolysis	BAK22096
**Ly212 R**	TGATGGGTCGTTATGCAACAG	59				
**Ly215 F**	ACCTGCTGGAACAGTTTGGA	58	1.92	122	competence/damage-inducible protein CinA	BAK20777
**Ly215 R**	GCGCTAAGCCTTGCATAACA	58				
**Ly231 F**	TCAGCATAATCTGGATCTGTAGCA	62	2.24	113	N-acetylmuramoyl-L-alanine amidase	BAK21795
**Ly231 R**	GGTGTTAGTTGGAATCCTCGAC	62				
**Ly334 F**	TGACACACCAAGGATGGTTCC	61	2.00	101	hypothetical protein	BAK21898
**Ly334 R**	TTGGTGTTGTTGGTCAGGCT	58				
**Ly952 F**	CAGAGGACAGAGGAATCGCA	58	2.08	111	LysM domain protein	BAK21514
**Ly952 R**	TTGTGGTCCCTCGCCATTTT	58				
**pgh133 F**	GGGCTGCATTGGTTGAAGGA	60	2.01	103	secreted antigen GbpB/SagA/PcsB, putative peptidoglycan hydrolase	BAK20695
**pgh133 R**	ATCACAGATTCTCCCGGTGC	60				
**atlA1 F**	AACCGGGCACGAATCAAGAA	58	1.99	144	secreted endo-beta-N-acetylglucosaminidase EndoS	BAK20932
**atlA1 R**	GGGTGGGTGTTTGTTGC	58				

Table S5: Results of pairwise comparison Dunn's tests with Bonferroni corrected p-values for the autolysis experiment. (significant differences are highlighted in italic p-values)

Comparison	z-value	P (adjusted)
119 vs. 119 - 119	0.67	1.00
119 vs. 119 - 49.3	3.84	*0.0019*
119 - 119 vs. 119 - 49.3	3.54	*0.006*
119 vs. 49.3	10.76	< *0.0001*
119 – 119 vs. 49.3	11.10	< *0.0001*
119 - 49.3 vs. 49.3	7.97	< *0.0001*
119 vs. 49.3 - 119	11.31	< *0.0001*
119 - 119 vs. 49.3 - 119	11.89	< *0.0001*
119 - 49.3 vs. 49.3 - 119	8.35	< *0.0001*
49.3 vs. 49.3 - 119	-0.59	1.00
119 vs. 49.3 - 49.3	10.00	< *0.0001*
119 - 119 vs. 49.3 - 49.3	10.43	< *0.0001*
119 - 49.3 vs. 49.3 - 49.3	6.89	< *0.0001*
49.3 vs. 49.3 - 49.3	-1.89	0.89
49.3 - 119 vs. 49.3 - 49.3	-1.47	1.00

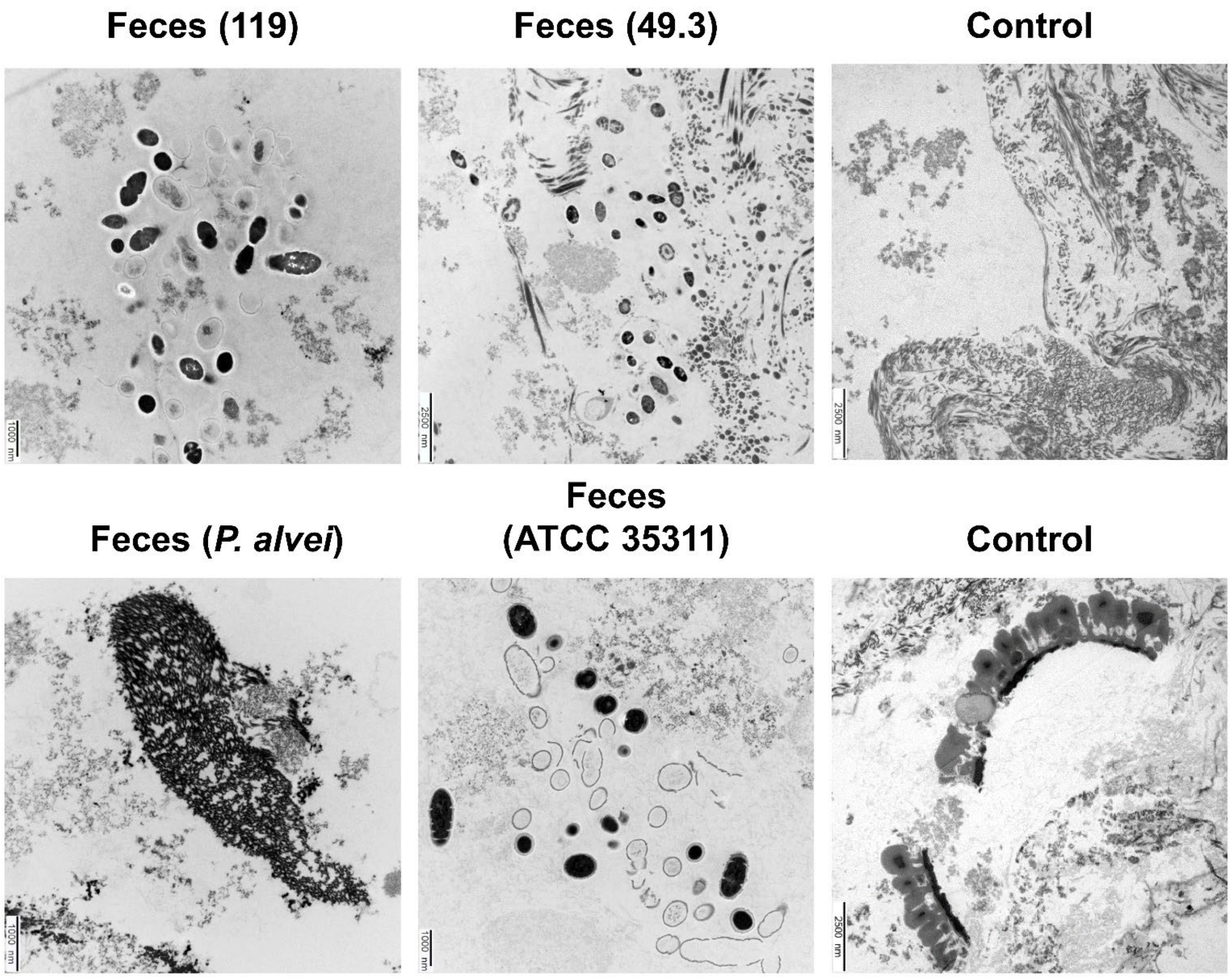

Figure S1: Transmission electron microscopy pictures of larvae faeces samples, larvae infected with *M. plutonius* (strain 49.3, 119, ATCC 35311) or feed with *P. alvei* only, and respective uninfected controls (free of bacteria). Bacteria are surrounded by food remains (mainly royal jelly) and larval gut lining and tissue.

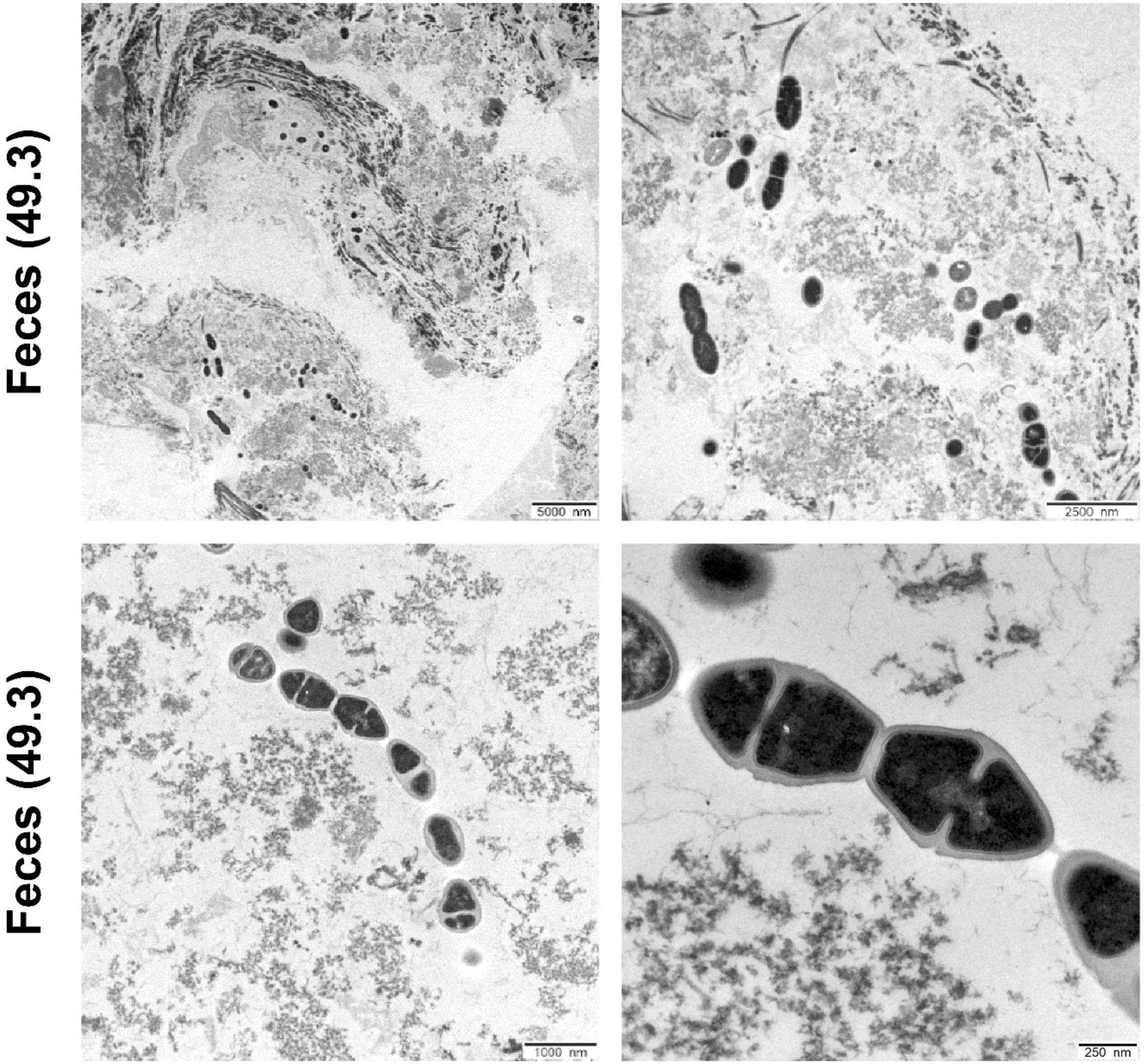

Figure S2: Transmission electron microscopy pictures of larvae faeces samples, larvae infected with *M. plutonius* (strain 49.3) only, at four different magnifications.

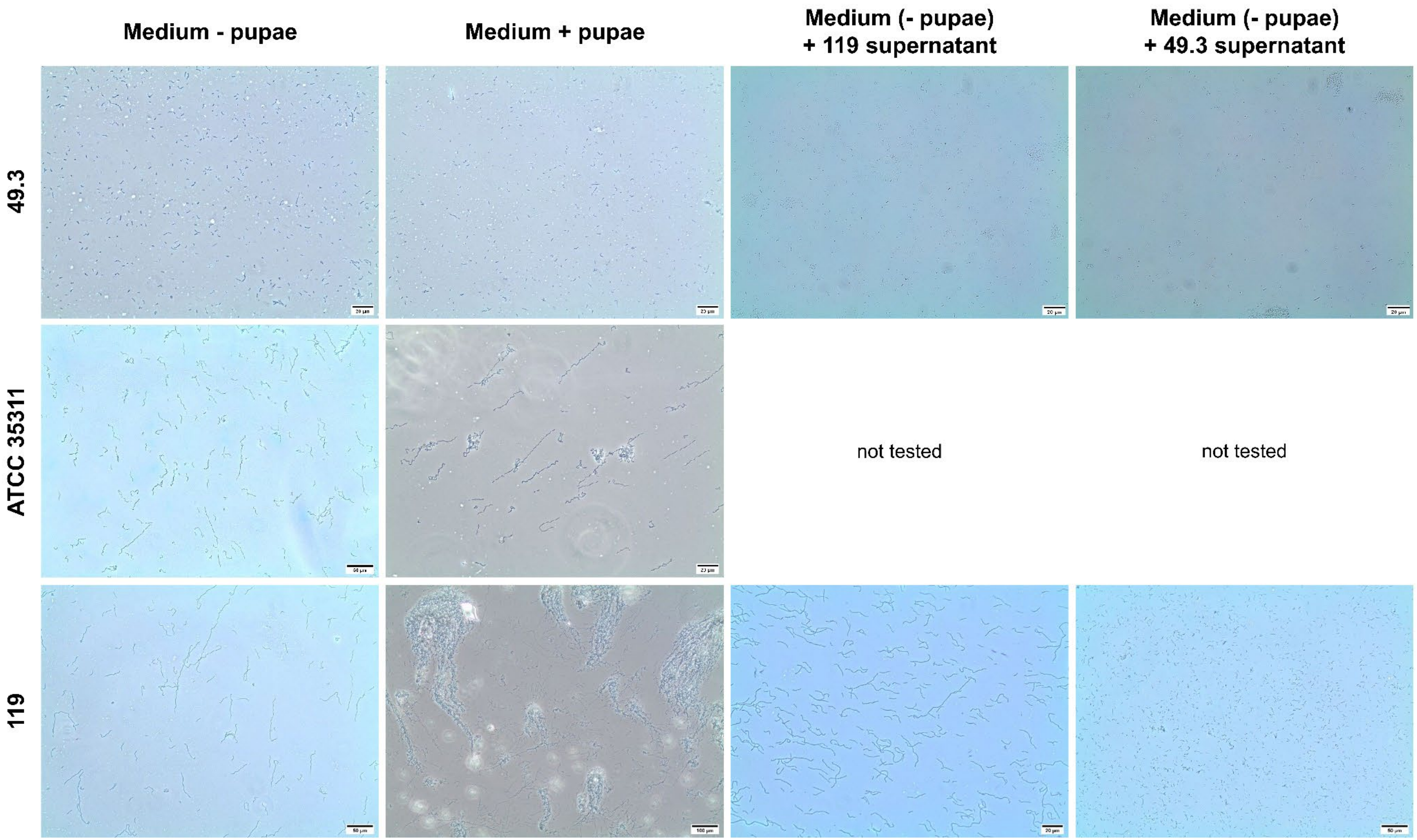

Figure S3: Light microscopy of *M. plutonius* growth assays in medium with or without homogenized honey bee drone pupae (white eye) (Lewkowski & Erler 2019), and strains 49.3 and 119 grown in medium containing the supernatant of the same strain (+ 49.3 / + 119) or medium containing the supernatant of the opposite strain (+ 119 / + 49.3).

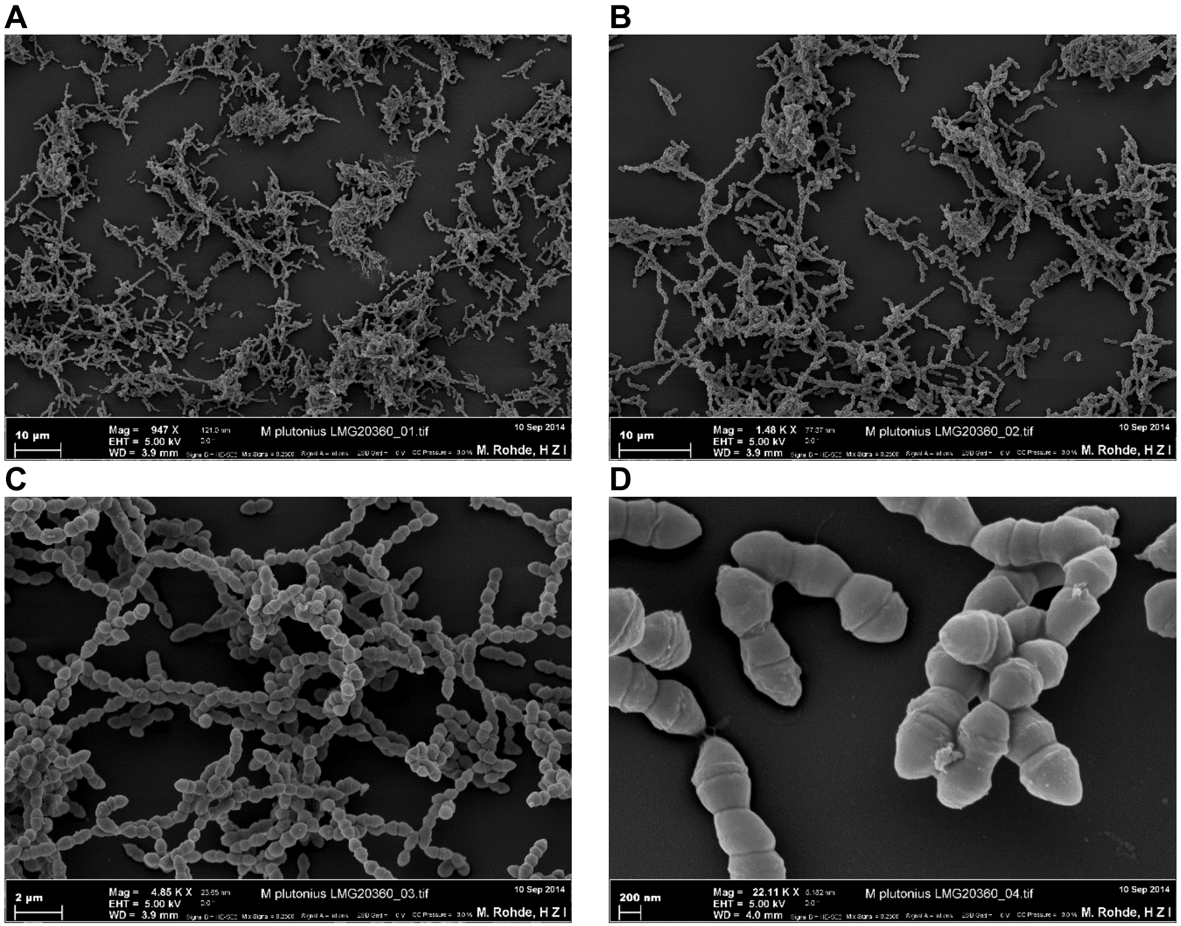

Figure S4: Scanning electron microscopy pictures of *Melissococcus plutonius* strain LMG20360 (= DSM 29964, ATCC 35311), at four different magnifications (A-D) (copy right by Hannes Beims and Manfred Rohde).
